# China runoff-field forecasting based on cross-scale gating and basin-topology attention

**DOI:** 10.1371/journal.pone.0350218

**Published:** 2026-06-22

**Authors:** Aiju Li, Xiang He, Kai Yang, Weiya Ge, Jian Hua

**Affiliations:** 1 Institute of Resource Survey and Evaluation, Jiangsu Geological Bureau, China; 2 Jiangsu New Energy Geological Survey Brigade, Nanjing, China; 3 Jiangsu Mineral Geological Survey Brigade, Nanjing, China; 4 Nanjing Geological Survey Center, China Geological Survey, Nanjing, China; Swedish Meteorological and Hydrological Institute, SWEDEN

## Abstract

Accurate multi-step runoff forecasting over China is important for flood control, water-resource management, and regional hydrological assessment. However, existing data-driven methods often struggle to jointly capture temporal variations at different time scales and the directional hydrological dependencies imposed by river networks, which limits forecasting accuracy and spatial structural consistency. To address this issue, this paper proposes a spatiotemporal forecasting framework that combines cross-scale temporal fusion with basin-topology-guided spatial modeling. Specifically, a multi-scale temporal module with cross-scale gating is introduced to adaptively integrate short-, medium-, and long-term runoff variations, while a basin-topology attention module incorporates upstream–downstream connectivity into spatial dependency learning. Experiments are conducted on a China-scale gridded runoff forecasting benchmark derived from the publicly available GloFAS Historical dataset through spatial filtering, valid-region masking, and forecasting-oriented sample construction. The proposed method achieves better overall performance than representative baselines in terms of MAE, RMSE, PSNR, and SSIM. In the overall comparison, it reaches MAE 0.0269 and RMSE 0.0603 in normalized log-scale runoff units, PSNR 24.39, and SSIM 0.9273, while maintaining a moderate parameter size and practical inference efficiency. The results demonstrate that the proposed framework reduces numerical errors and better preserves the spatial patterns of runoff fields. Ablation studies further confirm that both cross-scale gating and basin-topology attention contribute consistently to the overall improvement.

## 1 Introduction

Spatiotemporal forecasting of runoff fields is a fundamental component of hydrological forecasting, flood-risk management, water-resources scheduling, and ecological security assessment [1–3]. Compared with forecasting discharge time series at individual gauging stations, runoff-field forecasting directly outputs a continuous spatially distributed field, which can simultaneously characterize heterogeneous responses across different river reaches and terrain units within a region, thereby providing finer-grained decision support for cross-basin coordinated operations, flood-evolution monitoring, and regional risk zoning [4–6]. With the ongoing accumulation of reanalysis products and global hydrological datasets, leveraging long-term, continuous gridded runoff data to perform data-driven multi-step forecasting over China not only helps improve rapid modeling of complex hydrological processes, but also offers a reproducible methodological basis for operationally deployable regional intelligent forecasting.

However, runoff-field forecasting over China faces pronounced challenges arising from structural complexity and non-stationarity [7]. On the one hand, runoff dynamics are jointly driven by seasonality, extreme precipitation, snowmelt, and human activities, exhibiting strong nonlinearity and distribution shifts, which makes multi-step forecasting errors prone to temporal accumulation. These processes involve both fast hydrological responses, such as rainfall-driven local surges and short-term routing fluctuations, and relatively slow variations associated with seasonal background changes, snowmelt evolution, and basin-scale flow propagation. On the other hand, runoff fields show clear spatial heterogeneity and slender-structure characteristics: major river networks and flow-concentration corridors are governed by directional propagation and upstream–downstream coupling [8,9]. Grid-based modeling purely relying on Euclidean-neighborhood convolutions or global attention often fails to explicitly capture such topological dependencies, leading to broken high-value bands, excessive smoothing, or biased local extremes [10]. Meanwhile, runoff dynamics also involve mixed temporal scales where short-term fluctuations and long-term trends coexist; previous studies [11,12] typically addressed this issue either by using recurrent models with a single effective temporal receptive field, or by enlarging temporal context through stacked sequence encoders and attention-based aggregation. Although such strategies can partially improve temporal representation, they usually do not explicitly model cross-scale interactions or dynamically control information exchange among different temporal resolutions. Without cross-scale interaction mechanisms, the selection and fusion of critical scales can become unstable, thereby degrading structural consistency and generalization. As a result, even when longer temporal context is incorporated, the model may still fail to distinguish which scale is more informative under different hydrological conditions, and the resulting representations remain insufficient for preserving both temporal adaptivity and spatial structural fidelity in runoff-field forecasting.

To address these issues, this paper proposes an end-to-end spatiotemporal forecasting framework that integrates a cross-scale gating mechanism with basin-topology attention. Compared with previous efforts that mainly enhance temporal encoding capacity, the proposed framework is designed to explicitly select, fuse, and regulate multi-scale temporal information while simultaneously incorporating basin topology into spatial dependency modeling. In this paper, “runoff-field forecasting” refers to the forecasting of spatially distributed runoff over a gridded geographic domain, rather than runoff estimation at a single station or isolated river reach. At the temporal modeling level, we introduce a multi-scale LSTM to represent dynamic patterns under different temporal granularities, and employ cross-scale gating to enable adaptive interaction and fusion across scales, allowing the model to dynamically emphasize the most informative scale cues under different forecasting conditions. At the spatial-structure level, we further incorporate basin-topology attention by injecting directional river-network priors and reachability-based path correlations into the attention weights, thereby explicitly strengthening upstream–downstream dependency modeling and connectivity characterization over key corridors. Through the synergistic effect of cross-scale dynamic fusion and topology-aware structural constraints, the model not only reduces numerical errors but also better preserves the spatial textures and structural coherence of runoff fields, improving the stability and reliability of multi-step forecasting.

The main contributions of this work are as follows:

(1) We propose a structured spatiotemporal modeling approach for multi-step runoff-field forecasting over China, which jointly models multi-scale temporal dynamics and upstream–downstream dependencies by unifying cross-scale gated temporal fusion with basin-topology attention as a spatial structural prior.
(2) We construct and curate a gridded runoff-field forecasting dataset over China, together with the corresponding valid-region mask and preprocessing pipeline, yielding continuous daily samples and a reproducible data-splitting protocol that can serve as a readily usable benchmark for related studies.
(3) Under a unified evaluation setting, we compare against multiple representative methods and further validate the effectiveness and complementarity of each component through ablation studies, error visualizations, transect-profile comparisons, and Grad-CAM interpretability analysis, demonstrating stable advantages of the proposed framework in both accuracy and structural fidelity.

## 2 Related work

### 2.1 Spatiotemporal field forecasting paradigms and representation learning

Spatiotemporal field forecasting aims to learn spatiotemporal dependencies from multi-source observations and historical sequences, and to generate future estimates of continuous or discrete fields. Its central difficulty lies in jointly characterizing long-range temporal correlations, complex spatial interactions, and non-stationary variations across different scales [13]. Mao et al. presented a systematic survey for spatiotemporal forecasting, organizing a unified modeling lineage from Transformers to foundation models. They highlighted that large-scale pretraining and general-purpose representation learning can improve generalization across regions and tasks, and pointed out that attention mechanisms play a crucial role in capturing long-distance dependencies and structured relations [14]. Wang et al. provided a comprehensive review of deep learning methods in spatiotemporal data mining, summarizing the differences among sequence modeling, convolutional spatiotemporal encoding, and graph-structured learning in terms of feature extraction and predictive stability, while emphasizing the importance of improving representation robustness under sparse observations and noisy perturbations [15]. Hamdi et al. further summarized open problems in spatiotemporal data mining from the perspectives of task definitions, data heterogeneity, and evaluation challenges, noting that scale inconsistency and distribution shifts induced by multi-source drivers can significantly affect model generalization and interpretability; therefore, stronger structural inductive biases and adaptive fusion mechanisms are required [16]. Taken together, these studies suggest that the unified paradigm for spatiotemporal field forecasting is evolving from isolated modeling components toward a synergy between representation learning and structure modeling, motivating the incorporation of stronger cross-scale interactions and dynamic weight allocation to accommodate complex spatiotemporal processes.

In terms of structured representations, spatiotemporal graph neural networks have become an important paradigm for linking irregular spatial structures with temporal evolution. Jin et al. surveyed spatiotemporal graph predictive learning in urban computing, summarizing graph convolution- and attention-based approaches to modeling spatiotemporal dependencies, and emphasizing adaptive edge-weight learning and spatiotemporal coupling mechanisms to capture dynamic interactions and long-range propagation [17]. Capone et al. conducted a systematic review of graph neural network based spatiotemporal forecasting, pointing out that multi-scale structural modeling and gated fusion can enhance the identification of key channels and critical regions in complex relational networks, and discussing practical issues related to scalable training and stable inference [18]. Meanwhile, for forecasting and simulation of continuous physical fields, research has begun to improve the characterization of complex dynamical processes through data-driven, field-level representation learning. Lam et al. proposed a learning-based framework for medium-range global weather forecasting, demonstrating the feasibility of directly learning spatiotemporal evolution laws with deep models [19]; Bi et al. further introduced three-dimensional neural networks to improve medium-range weather forecasting accuracy and strengthen the modeling of three-dimensional spatiotemporal structures [20]. From the broader perspective of operator learning, Yin et al. proposed a scalable, geometry-informed operator learning framework for partial differential equation solvers, which learns the mapping from input conditions to solution fields in a unified manner [21]. Jiao et al. proposed a learn-once operator learning approach to enable efficient generalization under limited data [22], and Kumar et al. introduced multi-task DeepONet to improve the efficiency and transferability of partial differential equation solving [23]. Overall, research on spatiotemporal field forecasting is advancing along two main lines, namely structured graph modeling and continuous-field operator learning, and is increasingly integrating mechanisms such as attention and gating within a unified representation learning framework to jointly capture cross-scale dependencies and complex structural relations.

### 2.2 Runoff field forecasting under basin hydrology and river-network topology

Runoff field forecasting not only requires modeling the rainfall–runoff generation and routing chain together with temporal lags, but also must confront practical constraints such as heterogeneous catchment underlying surfaces, non-stationarity under extreme events, and sparse observations. Therefore, related studies typically start from data-driven temporal modeling and progressively introduce stronger physical consistency and improved regional generalization. Kratzert et al. proposed an LSTM-based rainfall–runoff modeling approach, demonstrating that deep sequence models can learn the nonlinear responses and temporal dependencies of hydrological systems without explicitly specifying process equations [24]. Kratzert et al. subsequently developed a learning framework for large-sample catchment datasets, emphasizing that cross-basin training can capture both generalizable regularities and regional differences, thereby improving generalization to unseen basins [25]. In the context of extreme events, Frame et al. presented a deep learning analysis for rainfall–runoff forecasting during extreme flood processes, pointing out that peak-period errors and tail risks impose stricter robustness requirements; hence, predictive mechanisms are needed that can maintain reliable representations under event-driven changes [26]. In addition, Li et al. investigated regionalization for global-scale hydrological deep learning models, showing that representation transformations from physical descriptors to stochastic vectors can affect cross-region transfer and generalization, which reflects the importance of representation design and regional heterogeneity modeling in runoff forecasting [27].

Beyond the temporal dimension, spatial dependencies within a catchment exhibit pronounced directionality and structural characteristics: upstream tributary inflows and river network connectivity determine information propagation routes and influence strengths, so relying solely on Euclidean proximity often fails to capture the true hydrological coupling. Sun et al. proposed a graph neural network approach for learning at the catchment-scale river network level, emphasizing that integrating physically connected river network structures with multi-source data helps learn spatial interactions that better align with mechanisms under channel propagation and routing constraints [28]. Liu et al. proposed a Bayesian graph neural network framework for daily runoff forecasting, systematically assessing the contribution of spatial connectivity to forecasting and improving reliability under structural dependence and observational noise through uncertainty modeling [29]. Furthermore, Wang et al. proposed a mass-conserving MC-LSTM model and evaluated it over the continental United States, indicating that incorporating conservation constraints can mitigate long-term simulation drift and improve hydrological consistency [30]. Wang et al. also studied accelerating lead times for intelligent flood forecasting by leveraging river network topology, showing that explicitly using topological information can strengthen the modeling of upstream-to-downstream propagation relations, thereby improving warning timeliness and predictive performance [31]. Overall, research on runoff fields is moving from purely temporal forecasting toward spatiotemporal joint modeling under structural constraints, and is increasingly integrating river network topology as a key inductive bias into mechanisms such as attention and gating, so as to better support cross-scale expressions of runoff generation and routing processes and stable generalization.

## 3 Method

### 3.1 Problem definition

This paper formalizes runoff field forecasting in China as a spatiotemporal sequence forecasting problem with watershed-structure priors. Let the study region be discretized into a regular grid of size H×W, where the runoff field at time *t* is denoted as $S_{t}\in {\mathbb{R}}^{H\times W}$. A binary mask $M\in \{0,1{\}}^{H\times W}$ is used to indicate valid grid cells wi*t*hin the watershed. Given a historical observation window of length *P*, ${𝒳}_{t}=\{S_{t-P+1},\ldots ,S_{t}\}$, the goal is to predict a future *Q*-step runoff field sequence ${𝒴}_{t}=\{S_{t+1},\ldots ,S_{t+Q}\}$. To explicitly characterize hydrological connectivity and upstream-to-downstream propagation, we abstract the watershed as a directed graph 𝒢=(𝒱,ℰ), where the node set 𝒱={1,…,N} represents river reaches or sub-basin units, the edge set ℰ encodes topological connectivity, and a weighted adjacency matrix $A\in {\mathbb{R}}^{N\times N}$ describes connection strengths. The model learns a parameterized mapping $f_{\theta }$ that outputs the predicted sequence by fusing spatiotemporal observations with topological priors, as


$$\begin{array}{c} {\hat{𝒴}}_{t}=f_{\theta }({𝒳}_{t},𝒢),\;{\hat{𝒴}}_{t}=\{{\hat{S}}_{t+1},\ldots ,{\hat{S}}_{t+Q}\}. \end{array}$$ (1)

During training, the parameters θ are learned by minimizing the forecasting error over valid regions. For example, a masked mean squared error objective can be adopted as


$$\begin{array}{c} \min_{\theta }\sum_{t}\sum_{\tau =1}^{Q}{\left\|M⊙({\hat{S}}_{t+\tau }-S_{t+\tau })\right\|}_{2}^{2}, \end{array}$$ (2)

where ⊙ denotes element-wise multiplication and $\|\cdot {\|}_{2}$ is the Frobenius norm. In this way, the model yields spatiotemporally consistent forecasting of future runoff fields.

### 3.2 Overall model architecture

**Fig 1:**
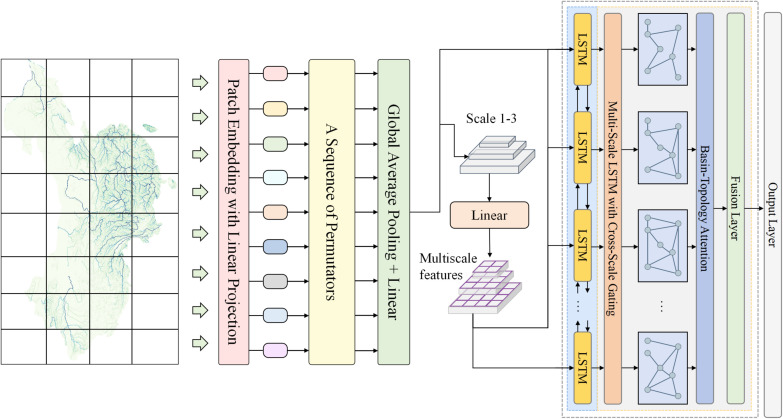
The figure illustrates the overall framework proposed in this paper for multi-step spatiotemporal forecasting of runoff fields in China. A Vision Transformer is adopted as the spatial encoding backbone, which performs patch embedding over the historical gridded runoff sequence and extracts multi-scale spatial representations. The resulting representations are then fed into a Multi-Scale LSTM with Cross-Scale Gating to model cross-scale temporal dependencies, while a Basin Topology Attention module is introduced to fuse river-network topology constraints. Finally, the fusion and output layers generate the predicted future runoff fields.

Based on the historical window ${𝒳}_{t}=\{S_{t-P+1},\ldots ,S_{t}\}$ and the watershed topological prior 𝒢 defined in the problem formulation, this paper builds an end-to-end spatiotemporal field forecasting framework. The key idea is to first employ a Vision Transformer to efficiently represent gridded runoff fields, and then perform joint multi-step inference of future runoff fields via cross-scale temporal modeling and topology-constrained attention [32]. Concretely, for each runoff field snapshot $S_{t^{\prime }}\in {\mathbb{R}}^{H\times W}$, we partition it into p×p patches and apply a linear projection to obtain $L=\frac{H}{p}\cdot \frac{W}{p}$ token representations. These tokens are then fed into a ViT(Vision Transformer) backbone to learn spatial correlations and global dependencies, producing a patch-level feature sequence $E_{t^{\prime }}\in {\mathbb{R}}^{L\times d}$, which compresses the original gridded field into a structured high-dimensional representation. This spatial encoding process can be written in a unified form as


$$\begin{array}{c} E_{t^{\prime }}=\Phi_{ViT}\;\left(Patch(S_{t^{\prime }})W_{e}+b_{e}\right),\;t^{\prime }\in [t-P+1,\;t], \end{array}$$ (3)

where Patch(·) denotes the patch unfolding operator, $W_{e}\in {\mathbb{R}}^{p^{2}\times d}$ and $b_{e}\in {\mathbb{R}}^{d}$ are the parameters of the linear projection, *d* denotes the embedding dimension of each patch token, and $\Phi_{ViT}(\cdot )$ is the ViT backbone. To form a compact sample-level representation, we further perform global aggregation over the patch dimension and apply a linear mapping to obtain a temporal feature vector $h_{t^{\prime }}\in {\mathbb{R}}^{d}$, which is jointly used by the subsequent temporal and structural modules. Specifically, the aggregated temporal feature is defined as


$$\begin{array}{c} h_{t^{\prime }}=W_{h}\;Pool(E_{t^{\prime }})+b_{h}, \end{array}$$ (4)

where Pool(·) denotes global aggregation over the patch dimension, and $W_{h}$ and $b_{h}$ are learnable mapping parameters. This design ensures consistency with the mapping form in the problem definition, i.e., ${\hat{𝒴}}_{t}=f_{\theta }({𝒳}_{t},𝒢)$. The overall architecture of the model is also presented here, as shown in Fig 1.

In the temporal modeling stage, we introduce a Multi-Scale LSTM with Cross-Scale Gating to capture both short-term perturbations and long-range evolution. By adaptively fusing information across different temporal scales through cross-scale gates, the model maintains stable representations under non-stationary hydrological processes. Specifically, for a scale index set 𝒦={1,…,K}, where *K* denotes the number of temporal scales and *K* = 3 in this study, the three adopted scales correspond to temporal receptive fields of 1, 3, and 7 time steps, respectively, the multi-scale LSTM produces a hidden-state sequence $\left\{u_{t^{\prime }}^{\left(k\right)}\right\}$ at each scale *k*, and selectively injects cross-scale information via a gating vector $g_{t^{\prime }}^{\left(k\right)}$, yielding a fused temporal representation $z_{t^{\prime }}$. We then inject watershed topology into the forecasting process using the graph structure 𝒢, and propose Basin-Topology Attention to perform attention-weighted propagation along river-network connectivity [33]. In this way, the directional upstream-to-downstream influence is explicitly modeled in the representation space and fused with the multi-scale temporal representation to form the final predictive encoding. The overall joint modeling of temporal dynamics and topology can be summarized as


$$\begin{array}{c} {\hat{𝒴}}_{t}=\Psi \;\left(BTA\;\left(MSLSTM\text{-}CSG\;\left(\{h_{t-P+1},\ldots ,h_{t}\}\right),\;𝒢\right)\right), \end{array}$$ (5)

where MSLSTM-CSG(·) denotes the multi-scale LSTM module with cross-scale gating, BTA(·,𝒢) denotes the attention module guided by watershed river-network topology, and Ψ(·) is the fusion and output mapping function used to generate the future *Q*-step runoff field sequence ${\hat{𝒴}}_{t}=\{{\hat{S}}_{t+1},\ldots ,{\hat{S}}_{t+Q}\}$. This design combines the global spatial modeling capability of ViT with cross-scale temporal dependencies and river-network topology constraints, enabling multi-step, spatiotemporally consistent runoff field forecasting within a unified framework.

### 3.3 Multi-scale LSTM with cross-scale gating

**Fig 2:**
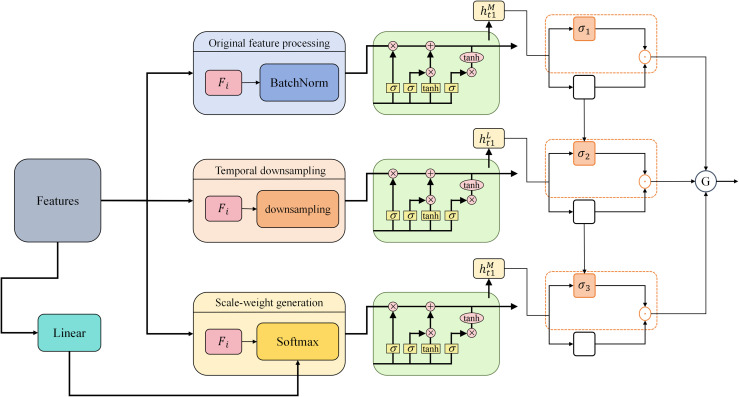
A schematic illustration of the Multi-Scale LSTM with Cross-Scale Gating module. The original features are processed at multiple temporal scales and fed into scale-specific LSTM encoders, and the hidden states from different scales are then adaptively weighted and fused using cross-scale gating coefficients to form a unified temporal representation for subsequent forecasting.

To jointly capture the fast responses induced by short-term rainfall disturbances and the slowly varying trends caused by mid-to-long-term routing and propagation in runoff field forecasting, we build a multi-scale temporal modeling module on top of the ViT-derived temporal representations $\left\{h_{t-P+1},\ldots ,h_{t}\right\}$. In the experiments, we use three temporal scales to model runoff dynamics, corresponding to short-, medium-, and long-range temporal dependencies. These scales are defined by different temporal receptive fields over the same historical window, with scale settings of 1, 3, and 7 time steps, respectively. The overall architecture of the module is also shown in Fig 2.

Let $h_{\tau }\in {\mathbb{R}}^{d}$ denote the feature vector at time τ obtained by spatial encoding and global aggregation. Multi-scale modeling constructs scale-specific feature streams by applying different temporal scale transformations to the same historical window. Let the scale set be 𝒮={1,…,S}, where each scale *s* corresponds to a temporal resolution or receptive field. In this study, smaller scales focus on recent short-term variations, whereas larger scales capture smoother and longer-range temporal evolution patterns. We adopt a differentiable scale transformation operator ${𝒯}_{s}(\cdot )$ to map the original sequence into a scale-specific sequence:


$$\begin{array}{c} x_{\tau }^{\left(s\right)}={𝒯}_{s}\;{\left(\{h_{\tau }{\}}_{\tau =t-P+1}^{t}\right)}_{\tau }\in {\mathbb{R}}^{d_{s}},\;s\in 𝒮, \end{array}$$ (6)

where $x_{\tau }^{\left(s\right)}$ is the input feature aligned to time τ under scale *s*, $d_{s}$ is the feature dimension at that scale, and ${𝒯}_{s}(\cdot )$ can be implemented as a combination of downsampling average pooling and a linear mapping to emulate hydrological responses over different time spans. Accordingly, the three scales respectively characterize rapid local fluctuations, intermediate transition dynamics, and relatively stable long-term tendencies. By explicitly constructing multi-scale inputs, the model can encode multi-timescale processes in parallel within a unified framework, providing a basis for subsequent cross-scale interactions.

For each scale *s*, we employ an LSTM [34] unit with normalization to recurrently encode the scale sequence and obtain the hidden states at that scale. Let $u_{\tau }^{\left(s\right)}\in {\mathbb{R}}^{m}$ and $c_{\tau }^{\left(s\right)}\in {\mathbb{R}}^{m}$ denote the hidden state and memory cell of scale *s* at time τ, where *m* is the hidden dimension. The within-scale recurrence is written as


$$\begin{array}{c} (u_{\tau }^{\left(s\right)},c_{\tau }^{\left(s\right)})={LSTM}_{s}\;(x_{\tau }^{\left(s\right)},u_{\tau -1}^{\left(s\right)},c_{\tau -1}^{\left(s\right)}),\;s\in 𝒮, \end{array}$$ (7)

where ${LSTM}_{s}(\cdot )$ uses scale-specific parameters, ensuring that different temporal scales can learn differentiated dynamic patterns. Since distributional discrepancies across scales may lead to unstable training, we first apply an affine transformation and batch normalization to the scale inputs [35], denoted as ${\bar{x}}_{\tau }^{\left(s\right)}={BN}_{s}(W^{\left(s\right)}x_{\tau }^{\left(s\right)}+b^{\left(s\right)})$, to reduce inter-scale magnitude differences that could interfere with gating and memory updates, thereby improving robustness and convergence of multi-scale modeling.

Within-scale encoding alone is still insufficient to explicitly express complementary relationships across scales. For example, shorter scales are more sensitive to abrupt local changes, whereas longer scales are better at providing a smooth trend prior. To this end, we propose Cross-Scale Gating, which introduces a learnable selective cross-scale injection mechanism at the scale level, enabling each scale to retrieve and fuse the most relevant information from other scales when updating its representation. More specifically, the information flow between scales is realized in two steps. First, the hidden states from all temporal scales at the same time step are aggregated into a shared cross-scale context vector, which summarizes complementary information from different receptive fields. Second, this shared context is selectively injected back into each scale through a scale-specific gating vector, so that each scale can adaptively absorb useful information from the other scales while preserving its own temporal characteristics. We first aggregate the multi-scale hidden states at the same time τ to form a cross-scale context vector:


$$\begin{array}{c} g_{\tau }=\phi \;\left(\sum_{r\in 𝒮}\alpha_{\tau }^{\left(r\right)}\;u_{\tau }^{\left(r\right)}\right)\in {\mathbb{R}}^{m},\;\alpha_{\tau }^{\left(r\right)}=\frac{\exp(w_{g}^{⊤}u_{\tau }^{\left(r\right)})}{\sum_{k\in 𝒮}\exp(w_{g}^{⊤}u_{\tau }^{\left(k\right)})}, \end{array}$$ (8)

where $\alpha_{\tau }^{\left(r\right)}$ is the importance weight of scale *r* at time τ, $w_{g}\in {\mathbb{R}}^{m}$ is a learnable vector, and ϕ(·) is a nonlinear activation function used to form a shared semantic context across scales. This context reflects complementary explanations of the current dynamics from different temporal scales and provides a unified information source for subsequent gated injection.

Based on the cross-scale context $g_{\tau }$, we construct a scale-specific injection gate for each scale *s* to control the strength and direction with which cross-scale information enters that scale representation, thereby avoiding mutual interference across scales. Specifically, we define a cross-scale gating vector $r_{\tau }^{\left(s\right)}\in (0,1{)}^{m}$ and obtain the gated fused hidden state ${\tilde{u}}_{\tau }^{\left(s\right)}$ as


$$\begin{array}{c} r_{\tau }^{\left(s\right)}=\sigma \;\left(W_{r}^{\left(s\right)}[u_{\tau }^{\left(s\right)};g_{\tau }]+b_{r}^{\left(s\right)}\right),\;{\tilde{u}}_{\tau }^{\left(s\right)}=u_{\tau }^{\left(s\right)}+r_{\tau }^{\left(s\right)}⊙g_{\tau }, \end{array}$$ (9)

where [·;·] denotes vector concatenation, $W_{r}^{\left(s\right)}\in {\mathbb{R}}^{m\times 2m}$ and $b_{r}^{\left(s\right)}\in {\mathbb{R}}^{m}$ are the gating parameters for scale *s*, σ(·) is the sigmoid function, and ⊙ denotes element-wise multiplication. This design allows scale *s* to preserve its own dynamic characteristics while adaptively absorbing complementary evidence from other scales, which is particularly suitable for runoff processes where responses at different time scales are superposed and mutually modulated.

Finally, to provide a single yet information-rich temporal representation for the subsequent Basin-Topology Attention module, we fuse the gated hidden states across scales to obtain the final temporal feature $z_{\tau }\in {\mathbb{R}}^{m}$. Since the importance of different scales varies over time, we further introduce time-dependent scale fusion weights $\beta_{\tau }^{\left(s\right)}$ and aggregate multi-scale representations into a unified encoding:


$$\begin{array}{c} z_{\tau }=\sum_{s\in 𝒮}\beta_{\tau }^{\left(s\right)}\;{\tilde{u}}_{\tau }^{\left(s\right)},\;\beta_{\tau }^{\left(s\right)}=\frac{\exp(w_{f}^{⊤}{\tilde{u}}_{\tau }^{\left(s\right)})}{\sum_{k\in 𝒮}\exp(w_{f}^{⊤}{\tilde{u}}_{\tau }^{\left(k\right)})}, \end{array}$$ (10)

where $w_{f}\in {\mathbb{R}}^{m}$ is a learnable vector and $\beta_{\tau }^{\left(s\right)}$ denotes the contribution of scale *s* during fusion at time τ. The resulting sequence $\left\{z_{t-P+1},\ldots ,z_{t}\right\}$ retains the short-scale sensitivity to rapid variations, inherits the long-scale robust characterization of global trends, and enables selective information flow through cross-scale gating, thereby establishing a unified temporal representation foundation for subsequent attention propagation with river-network topology constraints.

### 3.4 Basin-topology attention

**Fig 3:**
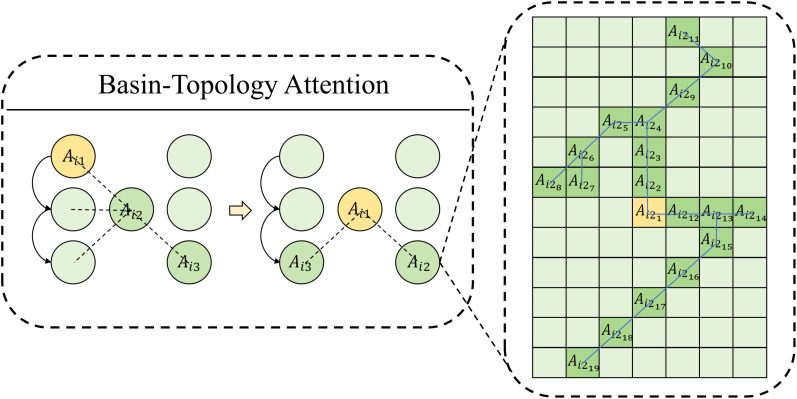
Illustration of the Basin-Topology Attention mechanism. The left part shows attention propagation on the directed river-network graph, where yellow denotes the target node, green denotes topologically related nodes, and light-colored nodes are not involved in the current propagation step. The right part maps these associations back to the runoff grid, where yellow marks the target location and green marks locations with valid topological influence. This figure illustrates the core idea of Basin-Topology Attention: under the constraints of the directed river-network topology, attention-weighted information propagation is performed along upstream-to-downstream reachable paths, and the resulting topological associations are mapped back to the grid space to enhance the structural consistency of runoff field forecasting.

After obtaining the unified temporal representation sequence $\left\{z_{t-P+1},\ldots ,z_{t}\right\}$ from the Multi-Scale LSTM with Cross-Scale Gating module, we further inject the directional connectivity of the watershed river network as a structural prior into the forecasting process, and propose Basin-Topology Attention to explicitly characterize upstream-to-downstream routing propagation and the non-local dependencies induced by tributary confluences. The river-network topology is derived from the spatial connectivity structure of the runoff field itself by abstracting hydrologically connected river reaches or sub-basin units as graph nodes and organizing their flow-direction relationships into a directed graph. In this graph, an upstream–downstream relationship is defined according to the directed runoff propagation path, that is, node *u* is regarded as upstream of node *v* if runoff information can propagate from *u* to *v* along a valid directed path. The overall architecture of the module is also shown in Fig 3.

Let the watershed topology be abstracted as a directed graph 𝒢=(𝒱,ℰ), where 𝒱={1,…,N} denotes the set of river-reach or sub-basin nodes, ℰ denotes the set of directed edges, and $A\in {\mathbb{R}}^{N\times N}$ is the weighted adjacency matrix, with $A_{uv}>0$ indicating the hydrological connectivity strength from node *u* to node *v*. Here, the adjacency matrix *A* characterizes direct first-order hydrological connections, whereas the corresponding reachability structure characterizes whether two nodes are connected through one or multiple valid downstream propagation paths. To align the temporal representation with topological nodes, we introduce at each time step τ a learnable projection operator that maps $z_{\tau }\in {\mathbb{R}}^{m}$ to a node-feature matrix $X_{\tau }\in {\mathbb{R}}^{N\times d_{g}}$:


$$\begin{array}{c} X_{\tau }=\Pi (z_{\tau })=reshape\;\left(z_{\tau }W_{\Pi }+b_{\Pi },\;N,\;d_{g}\right),\;\tau \in [t-P+1,\;t], \end{array}$$ (11)

where $W_{\Pi }\in {\mathbb{R}}^{m\times (Nd_{g})}$ and $b_{\Pi }\in {\mathbb{R}}^{Nd_{g}}$ are learnable parameters, and $d_{g}$ is the graph feature dimension. This alignment enables topology-constrained information propagation in the river-network node space, while keeping the input form consistent with the problem definition ${\hat{𝒴}}_{t}=f_{\theta }({𝒳}_{t},𝒢)$.

Since river-network propagation exhibits hierarchical path structures, and the influence of a node on downstream regions is often transmitted stepwise along trunk paths and superposed at confluences, we explicitly introduce a topology-path-based structural bias into attention computation [36]. Let 𝒫(u→v) denote the set of reachable paths from an upstream node *u* to a downstream node *v*. We define the attenuation weight of a path *p* as $\gamma^{\left|p\right|}$ (where |*p*| is the path length and γ∈(0,1) is a decay factor), and obtain the path-aware topological affinity as


$$\begin{array}{c} B_{uv}=\sum_{p\in 𝒫(u\to v)}\gamma^{\left|p\right|}\prod_{(i,j)\in p}A_{ij}, \end{array}$$ (12)

where $B_{uv}$ characterizes the aggregated propagation strength from *u* to *v*, reflecting both connectivity and distance-based attenuation. When only local first-order topology is needed, 𝒫(u→v) can degenerate into a single-edge set so that $B_{uv}$ is approximated by $A_{uv}$. Based on this structural affinity, we define a topology-constrained mask $M_{B}\in \{0,1{\}}^{N\times N}$, where $M_{B}(u,v)=1$ indicates $B_{uv}>0$, thereby restricting candidate attention relations to the river-network reachable domain and preventing non-physical cross-branch connections from being spuriously amplified. Therefore, the attention computation is constrained jointly by direct adjacency and multi-step reachability: direct adjacency preserves local hydrological continuity, while reachability further restricts information aggregation to physically meaningful upstream–downstream paths.

At each time step τ, we compute multi-head topology attention over node features to achieve adaptive, river-network-aware weighted aggregation. For the *r*-th attention head, we first construct the query, key, and value matrices:


$$\begin{array}{c} Q_{\tau }^{\left(r\right)}=X_{\tau }W_{Q}^{\left(r\right)},\;K_{\tau }^{\left(r\right)}=X_{\tau }W_{K}^{\left(r\right)},\;V_{\tau }^{\left(r\right)}=X_{\tau }W_{V}^{\left(r\right)}, \end{array}$$ (13)

where $W_{Q}^{\left(r\right)},W_{K}^{\left(r\right)},W_{V}^{\left(r\right)}\in {\mathbb{R}}^{d_{g}\times d_{h}}$ are learnable matrices and $d_{h}$ is the per-head dimension. We then compute attention energies within the topological reachable domain and inject the path affinity as a structural bias:


$$\begin{array}{c} e_{\tau }^{\left(r\right)}(u,v)=\frac{\left(Q_{\tau }^{\left(r\right)}(u){)}^{⊤}K_{\tau }^{\left(r\right)}(v\right)}{\sqrt{d_{h}}}+\lambda \;\log\;(B_{uv}+\epsilon ),\;M_{B}(u,v)=1, \end{array}$$ (14)

where λ controls the strength of the structural bias and ϵ>0 is a numerical stabilizer. This formulation makes attention weights driven by both feature similarity and river-network path propagation strength, aligning with the superposition behavior of runoff propagation along main stems and at tributary confluences. For node pairs that do not satisfy the topological reachability constraint, their attention scores are masked out before softmax normalization, so that invalid cross-branch or reverse-direction interactions do not participate in the aggregation process. For a fixed target node *v*, we apply softmax normalization over its topologically reachable upstream set $𝒰(v)=\{u|M_{B}(u,v)=1\}$ and aggregate to obtain the updated node representation:


$$\begin{array}{c} \alpha_{\tau }^{\left(r\right)}(u,v)=\frac{\exp\;(e_{\tau }^{\left(r\right)}(u,v))}{\sum_{u^{\prime }\in 𝒰(v)}\exp\;(e_{\tau }^{\left(r\right)}(u^{\prime },v))},\;{\tilde{X}}_{\tau }^{\left(r\right)}(v)=\sum_{u\in 𝒰(v)}\alpha_{\tau }^{\left(r\right)}(u,v)\;V_{\tau }^{\left(r\right)}(u), \end{array}$$ (15)

where $\alpha_{\tau }^{\left(r\right)}(u,v)$ denotes the contribution weight of upstream node *u* to node *v* at time τ, and ${\tilde{X}}_{\tau }^{\left(r\right)}(v)$ is the topology-weighted aggregated representation.

To account for multi-path information and complementarity across propagation scales, we concatenate multi-head outputs and apply a linear projection, and adopt a residual connection to preserve the dynamics provided by the cross-scale temporal module while improving training stability. The output of the topology attention layer is defined as


$$\begin{array}{c} {\hat{X}}_{\tau }=LN\;\left(X_{\tau }+Concat\;({\tilde{X}}_{\tau }^{\left(1\right)},\ldots ,{\tilde{X}}_{\tau }^{\left(R\right)})W_{O}\right), \end{array}$$ (16)

where *R* is the number of heads, $W_{O}\in {\mathbb{R}}^{(Rd_{h})\times d_{g}}$ is the output projection matrix, and LN(·) denotes layer normalization. Finally, to interface with the fusion and output layers in the overall architecture, we perform a global readout over the topology-enhanced node features to obtain a structure-aware temporal vector $b_{\tau }\in {\mathbb{R}}^{m}$ as the input for subsequent forecasting:


$$\begin{array}{c} b_{\tau }=\Omega ({\hat{X}}_{\tau })=\rho \;\left(\sum_{v=1}^{N}\pi_{v}\;{\hat{X}}_{\tau }(v)\right)W_{\Omega }+b_{\Omega }, \end{array}$$ (17)

where $\pi_{v}$ denotes the node readout weight satisfying $\sum_{v}\pi_{v}=1$ (which can be uniform or given by learnable softmax weights), ρ(·) is a nonlinear activation function, and $W_{\Omega }\in {\mathbb{R}}^{d_{g}\times m}$ and $b_{\Omega }\in {\mathbb{R}}^{m}$ are learnable parameters. The resulting sequence $\left\{b_{t-P+1},\ldots ,b_{t}\right\}$ further integrates river-network topological propagation patterns on top of cross-scale dynamic representations, providing topology-consistent and interpretable representational support for multi-step runoff field forecasting.

### 3.5 Training loss and optimization objective

After obtaining the predicted sequence ${\hat{𝒴}}_{t}=\{{\hat{S}}_{t+1},\ldots ,{\hat{S}}_{t+Q}\}$ that fuses cross-scale dynamic representations and river-network topology constraints, we adopt a supervision objective defined on the valid watershed region to drive end-to-end training. This objective encourages the model to focus spatially on runoff evolution within the watershed boundary and to maintain temporal consistency across multi-step forecasts. Let the ground-truth future sequence be ${𝒴}_{t}=\{S_{t+1},\ldots ,S_{t+Q}\}$, and let the valid-watershed mask be $M\in \{0,1{\}}^{H\times W}$. We first define a step-wise masked reconstruction error and accumulate it over the forecasting horizon to obtain the base loss:


$$\begin{array}{c} {ℒ}_{rec}=\sum_{\tau =1}^{Q}{\left\|M⊙({\hat{S}}_{t+\tau }-S_{t+\tau })\right\|}_{F}^{2}, \end{array}$$ (18)

where ⊙ denotes element-wise multiplication and $\|\cdot {\|}_{F}$ denotes the Frobenius norm. To further enhance the model’s capability to capture peak runoff behavior and regional heterogeneity, we introduce within the valid region a robust term that is more sensitive to large errors, and penalize per-pixel residuals using a Smooth-$\ell_{1}$ form:


$$\begin{array}{c} {ℒ}_{rob}=\sum_{\tau =1}^{Q}\sum_{i=1}^{H}\sum_{j=1}^{W}M_{ij}\;smooth\ell_{1}\;\left({\hat{S}}_{t+\tau }(i,j)-S_{t+\tau }(i,j)\right), \end{array}$$ (19)

where


$$\begin{array}{c} smooth\ell_{1}(x)=\left\{ \begin{array}{cc} \frac{1}{2}x^{2}, & |x|<1,\\ |x|-\frac{1}{2}, & \text{otherwise}. \end{array}\right. \end{array}$$ (20)

This robust term suppresses the excessive influence of noisy points while preserving sufficient gradient signals for critical errors such as flood peaks, thereby improving forecasting stability under extreme processes when combined with the aforementioned cross-scale gating and topology attention designs.

By combining the above two components, the overall optimization objective is defined as a weighted sum, and the model parameters θ are learned end-to-end:


$$\begin{array}{c} \theta^{*}=\text{arg}\min_{\theta }\;{𝔼}_{t}[{ℒ}_{rec}+\lambda \;{ℒ}_{rob}], \end{array}$$ (21)

where ${𝔼}_{t}[\cdot ]$ denotes the empirical expectation over time indices of the training samples, and λ is a balancing coefficient. During optimization, we update θ using first-order gradient-based stochastic methods, and employ mini-batch training under multi-step supervision to jointly drive the convergence of the ViT spatial encoder, the cross-scale gated temporal module, and the watershed topology attention module, thereby yielding spatiotemporally consistent forecasting of future runoff fields. In all experiments, the balancing coefficient λ was selected based on validation performance, the main hyperparameters were tuned on the validation set, and training was conducted with a cosine learning-rate schedule and early stopping to improve convergence stability and avoid overfitting.

## 4 Dataset introduction and evaluation metrics

### 4.1 Dataset introduction

**Fig 4:**
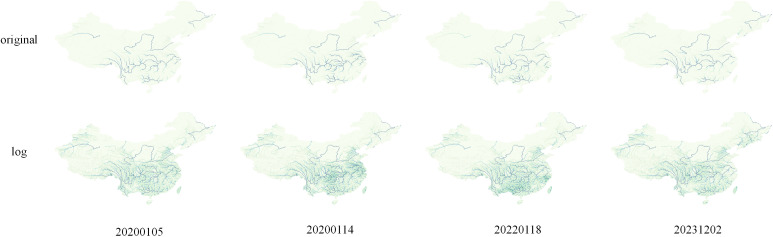
Comparison of raw data and data after log processing.

The runoff field data used in this study are obtained from the GloFAS Historical dataset [37] cems-glofas-historical provided by the Copernicus Climate Data Store. We select the historical reanalysis product with system_version set to version_4_0, hydrological_model set to lisflood, and product_type set to consolidated. The target variable is river_discharge_in_the_last_24_hours, which represents the river discharge statistic over the past 24 hours and can serve as a gridded representation of daily-scale runoff intensity. The original runoff grid follows the native spatial resolution provided by GloFAS Historical, and after cropping to the China region, the data are organized as daily gridded runoff fields for multi-step spatiotemporal forecasting. Accordingly, the temporal resolution used in this study is one day, which is consistent with the 24-hour accumulation property of the selected runoff variable. The temporal coverage spans from 2020-01-01–2025-11-01. The data are downloaded in NetCDF format by chunks and cached locally, forming a continuous daily time series that is used to construct the historical input window and the future multi-step supervision sequence for the spatiotemporal forecasting task. Following the forecasting setting adopted in this study, the historical input length is set to 30 days and the forecasting horizon is set to 7 days. The resulting daily samples are divided chronologically into training, validation, and testing subsets with a ratio of 7:1:2, in order to preserve the temporal ordering of hydrological evolution and avoid information leakage across periods. Since the runoff variable exhibits pronounced heavy-tailed distributions and large magnitude ranges across both space and time, we further apply a logarithmic transformation to the raw runoff values to mitigate the imbalance caused by extreme large values during training. The visualized runoff maps used in this study are author-generated from the GloFAS Historical NetCDF data, and no proprietary basemap, satellite screenshot, or third-party copyrighted map service is used in the figure preparation. Specifically, we use


$$\begin{array}{c} x_{\log}=\log_{10}(x+1), \end{array}$$ (22)

where *x* denotes the original runoff value. This transformation compresses the dynamic range, enhances the distinguishability of low-to-moderate flow regions, and improves numerical stability for model learning. After the logarithmic transformation, all samples are further normalized to the [0,1] range based on the statistics of the training set only, and the same normalization parameters are then applied to the validation and testing sets to ensure consistency. A comparison of the dataset before and after the log transform is shown in Fig 4.

**Fig 5:**
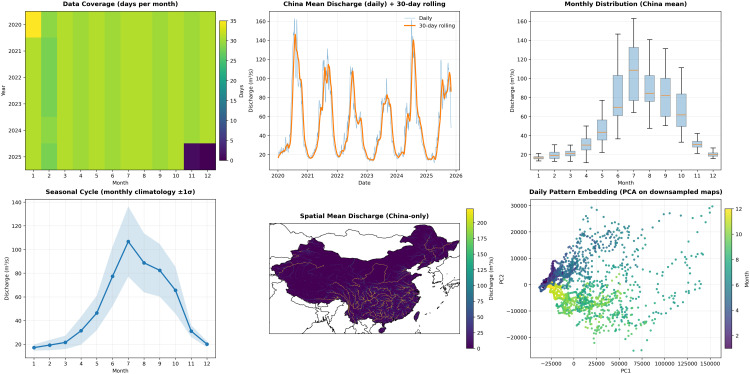
Overall statistical analysis results of the dataset.

For the spatial extent, we crop the data to the geographic range of China, with longitude $\left[73^{\circ },135^{\circ }\right]$ and latitude $\left[18^{\circ },54^{\circ }\right]$. We further extract the geometric outline of China based on Natural Earth administrative boundaries and construct a valid-region mask to remove grid cells outside the national boundary or otherwise invalid. Model training and inference take the cropped gridded sequence as the basic input and output. Meanwhile, during data preparation we keep both a linear-scale version and a log-scale version: the linear scale preserves the original physical semantics of the variable, while the log scale is used as the primary input representation to accommodate the heavy-tailed distribution and improve cross-region generalization. In addition, invalid grid cells and missing areas outside the effective national boundary are masked out throughout preprocessing, training, and evaluation, so that the forecasting benchmark remains spatially consistent with the valid-region constraint described in the overall framework. No additional temporal smoothing or manual filtering is introduced beyond cropping, masking, logarithmic transformation, and normalization. Under the same mask constraint, the two versions remain spatially consistent, providing a stable and coherent data foundation for the subsequent end-to-end runoff field forecasting framework based on ViT spatial encoding, cross-scale gated temporal modeling, and river-network topology attention. The overall statistical analysis of the dataset is shown in Fig 5.

Fig 5 presents the overall statistical characteristics of the constructed runoff-field dataset. The data coverage map indicates that the daily samples are generally continuous during the study period, while the low-coverage region at the end of 2025 is mainly caused by the data ending on 2025-11-01. The China-mean discharge curve, 30-day rolling average, monthly boxplots, and climatological seasonal cycle jointly reveal a clear annual pattern, with low runoff levels in winter and early spring and pronounced increases during the warm and wet seasons. The wider distributions in high-flow months further indicate stronger temporal variability and larger uncertainty during flood-season periods, highlighting the need to model both short-term fluctuations and longer-term seasonal evolution. The spatial mean discharge map shows strong regional heterogeneity, with high-value regions mainly distributed along major river-network corridors and wetter areas, suggesting that runoff fields are hydrologically structured rather than spatially homogeneous. In addition, the PCA embedding is used only for exploratory visualization by vectorizing each daily runoff field within the valid China mask and projecting it into a two-dimensional space. The resulting distribution shows clear seasonal organization, where low-flow months are relatively concentrated and wet-season samples are more dispersed, indicating that the dataset contains both stable seasonal background states and highly variable flood-season patterns. This observation further supports the necessity of cross-scale temporal modeling and basin-topology-aware spatial representation in runoff-field forecasting.

### 4.2 Evaluation metric

To evaluate the forecasting accuracy of future runoff fields, we adopt MAE and RMSE as pixel-wise error metrics, which measure the mean absolute deviation and the sensitivity to large errors, respectively. Both metrics are computed within the valid watershed region to avoid interference from invalid grid cells outside the watershed. Since the model is trained and evaluated on log-transformed and min–max normalized runoff fields, the reported MAE and RMSE values are expressed in normalized log-scale runoff units rather than in the original physical discharge unit.


$$\begin{array}{c} MAE=\frac{1}{\left|\Omega \right|}\sum_{i=1}^{H}\sum_{j=1}^{W}M_{ij}\left|{\hat{S}}_{t+\tau }(i,j)-S_{t+\tau }(i,j)\right| \end{array}$$ (23)

where $S_{t+\tau }\in {\mathbb{R}}^{H\times W}$ and ${\hat{S}}_{t+\tau }\in {\mathbb{R}}^{H\times W}$ denote the ground-truth and predicted runoff fields in the normalized log-scale space at the τ-th forecasting step, respectively, $M\in \{0,1{\}}^{H\times W}$ is the valid-region mask, and $|\Omega |=\sum_{i=1}^{H}\sum_{j=1}^{W}M_{ij}$ is the number of valid pixels.


$$\begin{array}{c} RMSE=\sqrt{\frac{1}{\left|\Omega \right|}\sum_{i=1}^{H}\sum_{j=1}^{W}M_{ij}{\left({\hat{S}}_{t+\tau }(i,j)-S_{t+\tau }(i,j)\right)}^{2}} \end{array}$$ (24)

where the squared term makes RMSE more sensitive to larger deviations in local flood peaks or abrupt-change regions, thereby providing a stricter reflection of forecasting errors under extreme processes. Accordingly, both MAE and RMSE in the experimental tables should be interpreted as errors measured on the normalized log-scale runoff fields.

In addition to the above error-based metrics, we further introduce Nash–Sutcliffe Efficiency (NSE) and Kling–Gupta Efficiency (KGE) as hydrology-oriented evaluation criteria to assess whether the predicted runoff fields can accurately reproduce the temporal variation process, fluctuation magnitude, and statistical consistency of the reference runoff. Since this study focuses on gridded runoff-field forecasting rather than single-station prediction, NSE and KGE are computed over the valid watershed region by first collecting the predicted and observed runoff values at all valid grid cells and forecasting steps, and then performing regional evaluation on the paired valid samples. In this way, the two metrics comprehensively reflect the overall hydrological consistency between the predicted fields and the ground-truth fields within the study area. NSE and KGE are dimensionless metrics.

The NSE metric evaluates the relative predictive skill of the model compared with the mean observation baseline, and is defined as


$$\begin{array}{c} NSE=1-\frac{\sum_{(i,j,\tau )\in \Omega^{*}}{\left({\hat{S}}_{t+\tau }(i,j)-S_{t+\tau }(i,j)\right)}^{2}}{\sum_{(i,j,\tau )\in \Omega^{*}}{\left(S_{t+\tau }(i,j)-\bar{S}\right)}^{2}} \end{array}$$ (25)

where $\Omega^{*}=\{(i,j,\tau )|M_{ij}=1\}$ denotes the set of all valid evaluation samples across the spatial region and forecasting horizon, and $\bar{S}$ is the mean of all ground-truth runoff values over $\Omega^{*}$. A larger NSE indicates better predictive skill, and a value closer to 1 implies that the predicted runoff fields more accurately capture the actual hydrological evolution process.

The KGE metric further evaluates prediction quality from three complementary aspects, namely linear correlation, variability ratio, and bias ratio, and is defined as


$$\begin{array}{c} KGE=1-\sqrt{(r-1{)}^{2}+(\alpha -1{)}^{2}+(\beta -1{)}^{2}} \end{array}$$ (26)

where


$$\begin{array}{c} r=\frac{\sum_{(i,j,\tau )\in \Omega^{*}}\left({\hat{S}}_{t+\tau }(i,j)-\mu_{\hat{S}}\right)\left(S_{t+\tau }(i,j)-\mu_{S}\right)}{\sqrt{\sum_{(i,j,\tau )\in \Omega^{*}}{\left({\hat{S}}_{t+\tau }(i,j)-\mu_{\hat{S}}\right)}^{2}}\sqrt{\sum_{(i,j,\tau )\in \Omega^{*}}{\left(S_{t+\tau }(i,j)-\mu_{S}\right)}^{2}}} \end{array}$$ (27)

$$\begin{array}{c} \alpha =\frac{\sigma_{\hat{S}}}{\sigma_{S}},\;\beta =\frac{\mu_{\hat{S}}}{\mu_{S}} \end{array}$$ (28)

with $\mu_{\hat{S}}$ and $\mu_{S}$ denoting the mean values of the predicted and ground-truth runoff over $\Omega^{*}$, respectively, and $\sigma_{\hat{S}}$ and $\sigma_{S}$ denoting the corresponding standard deviations. Here, *r* measures the linear consistency between prediction and observation, α characterizes the consistency of fluctuation magnitude, and β reflects the bias in the overall runoff level. A larger KGE indicates better overall agreement between the predicted and observed runoff fields in terms of correlation, variability, and bias.

To assess the quality of the predicted fields in terms of overall signal-to-noise ratio and spatial structural consistency, we further adopt PSNR and SSIM to measure fidelity and structural similarity. PSNR reflects the overall reconstruction quality based on mean squared error, while SSIM characterizes spatial morphological consistency from three aspects, namely luminance, contrast, and structure. PSNR and SSIM are also dimensionless evaluation metrics.


$$\begin{array}{c} PSNR=10\log_{10}\;\left(\frac{{MAX}^{2}}{MSE}\right),\;MSE=\frac{1}{\left|\Omega \right|}\sum_{i=1}^{H}\sum_{j=1}^{W}M_{ij}{\left({\hat{S}}_{t+\tau }(i,j)-S_{t+\tau }(i,j)\right)}^{2} \end{array}$$ (29)

where MAX denotes the upper bound of the pixel dynamic range used in evaluation, and MSE is the mean squared error computed over the valid region Ω.


$$\begin{array}{c} SSIM=\frac{\left(2\mu_{\hat{S}}\mu_{S}+c_{1})(2\sigma_{\hat{S}S}+c_{2}\right)}{\left(\mu_{\hat{S}}^{2}+\mu_{S}^{2}+c_{1})(\sigma_{\hat{S}}^{2}+\sigma_{S}^{2}+c_{2}\right)} \end{array}$$ (30)

where $\mu_{\hat{S}}$ and $\mu_{S}$ are the means of the predicted field and the ground-truth field, respectively, $\sigma_{\hat{S}}^{2}$ and $\sigma_{S}^{2}$ are the variances, $\sigma_{\hat{S}S}$ is the covariance, and *c*_1_ and *c*_2_ are stability constants to avoid zero denominators. A larger SSIM indicates higher spatial structural consistency.

### 4.3 Experimental setup

All experiments in this study were conducted under the same computing environment to ensure reproducibility and a fair comparison. The model was implemented in PyTorch and trained on a single GPU. The input sequence length was set to *P* = 30 (historical window), and the forecasting horizon was set to *Q* = 7. We adopted the AdamW optimizer with an initial learning rate of $1\times 10^{-4}$, a batch size of 8, and trained the model for 80 epochs. A cosine annealing learning-rate schedule with warmup was used to mitigate overfitting. To improve training stability, we enabled automatic mixed precision (AMP) and applied gradient clipping. For the Vision Transformer spatial encoder, each runoff field was partitioned into non-overlapping patches of size 16×16, resulting in a regular sequence of patch tokens for spatial representation learning. Under the spatial resolution used in this study, this configuration produced a fixed number of patches for each input sample, and no patch overlap was introduced. Learnable absolute positional embeddings were added to the patch embeddings to preserve the spatial arrangement of runoff patterns before they were fed into the Transformer encoder. These settings were kept unchanged in all experiments to ensure consistent spatial feature extraction across all compared methods using the same backbone. The key hardware/software specifications and core hyperparameters are summarized in Table 1.

**Table 1:** Hardware/software environment and main training hyperparameter settings.

Item	Configuration/Value
GPU	NVIDIA RTX 4090
CPU	Intel Xeon
Memory	64 GB
Operating System	Ubuntu 22.04 LTS
Python	3.10
Deep Learning Framework	PyTorch 2.1.0
CUDA / cuDNN	CUDA 12.1 / cuDNN 8.x
Input historical window	*P* = 30
Forecast horizon	*Q* = 7
Optimizer	AdamW
Initial learning rate	$1\times 10^{-4}$
Weight decay	$1\times 10^{-2}$
Batch size	8
Training epochs	80
Learning-rate scheduler	Cosine Annealing (warmup = 5 epochs)
Dropout	0.1
Gradient clipping	max_norm = 1.0
Patch size	16×16
Patch overlap	None
Positional encoding	Learnable absolute positional embedding
Loss weights	λ=1.0, γ=0.5
Acceleration strategy	AMP mixed-precision training

In all experiments, the models were implemented in Python with PyTorch and trained end-to-end under the same data split, preprocessing procedure, input length, forecasting horizon, and optimization setting to ensure a fair comparison across all methods. Unless otherwise required by the original architecture, the compared models were retrained using the same historical input of 30 days and the same 7-day forecasting target, and the reported quantitative results were obtained from the test set under this unified protocol. For the analysis figures and interpretation results, the error maps were constructed by calculating the pixel-wise absolute difference between the predicted runoff field and the ground truth, the transect-profile plots were generated by extracting runoff values along the same predefined spatial section from both forecasting and reference fields, and the Grad-CAM visualizations were produced by backpropagating the forecasting response to the last spatial feature layer and then overlaying the normalized activation maps onto the corresponding runoff fields. All these analyses were performed after model training using the test samples under the same experimental setting.

## 5 Experimental results and analysis

### 5.1 Experimental results compared with other models

To validate the overall effectiveness of the proposed method on multi-step runoff-field forecasting, we conduct comparative evaluations under the same data split and training configuration against representative spatiotemporal forecasting models, including 3DCNN [38], Strpm [39], Earthfarsser [40], Mlpst [41], UniST [42], Openstl [43], V2xpnp [44], and DFGNet [45]. The results are reported in the form of mean and standard deviation, obtained by training each model three times with different random seeds. We further report the number of parameters (Params) and inference speed (FPS) to assess the trade-off between accuracy and efficiency. The quantitative comparison results of all models are summarized in Table 2.

**Table 2:** Quantitative comparison with other models on the test phase.

Method	MAE	RMSE	PSNR	SSIM	NSE	KGE	Params	FPS
3DCNN	0.0418±0.0021	0.0837±0.0035	20.52±0.41	0.8812±0.0067	0.7941±0.0128	0.8126±0.0113	35.2M	62
Strpm	0.0395±0.0019	0.0791±0.0031	21.03±0.38	0.8895±0.0062	0.8214±0.0112	0.8368±0.0101	128.7M	37
Earthfarsser	0.0382±0.0020	0.0768±0.0030	21.42±0.36	0.8937±0.0059	0.8387±0.0107	0.8479±0.0098	142.5M	33
Mlpst	0.0367±0.0017	0.0739±0.0027	21.89±0.34	0.9026±0.0055	0.8516±0.0095	0.8643±0.0089	96.4M	48
Unist	0.0349±0.0016	0.0717±0.0026	22.31±0.31	0.9073±0.0051	0.8639±0.0091	0.8715±0.0084	121.8M	41
Openstl	0.0338±0.0015	0.0704±0.0024	22.57±0.28	0.9118±0.0047	0.8598±0.0088	0.8821±0.0080	150.9M	36
V2xpnp	0.0321±0.0014	0.0687±0.0023	22.89±0.27	0.9164±0.0044	0.8746±0.0083	0.8897±0.0076	89.3M	52
DFGNet	0.0306±0.0013	0.0659±0.0021	23.41±0.25	0.9221±0.0040	0.8912±0.0077	0.9018±0.0071	134.6M	39
Ours	0.0269±0.0011	0.0603±0.0019	24.39±0.22	0.9273±0.0038	0.9187±0.0069	0.9264±0.0063	117.3M	43

Table 2 shows that the proposed method achieves the best overall performance across all six evaluation metrics. Specifically, it yields the lowest MAE and RMSE, indicating stronger error control over runoff-field prediction, while its highest PSNR and SSIM further demonstrate better preservation of spatial quality and structural consistency. In addition, the proposed method attains the highest NSE and KGE, suggesting that it more accurately captures the overall hydrological variation, statistical consistency, and runoff fluctuation characteristics of the target fields. Although its parameter size and inference speed are not the most extreme among all compared methods, the model maintains a competitive computational cost while providing clear accuracy and hydrology-oriented evaluation advantages, which verifies the effectiveness of jointly introducing cross-scale temporal fusion and basin-topology-guided spatial modeling. Furthermore, to illustrate the stability and generalization, this paper presents the experimental results on the training set, as shown in Table 3.

**Table 3:** Training-phase quantitative comparison with other models.

Method	MAE	RMSE	PSNR	SSIM	NSE	KGE
3DCNN	0.0389±0.0017	0.0792±0.0028	21.07±0.46	0.8916±0.0059	0.8138±0.0105	0.8291±0.0124
Strpm	0.0368±0.0023	0.0754±0.0026	21.58±0.33	0.8979±0.0071	0.8397±0.0098	0.8526±0.0092
Earthfarsser	0.0357±0.0015	0.0731±0.0034	21.96±0.39	0.9035±0.0048	0.8542±0.0121	0.8613±0.0107
Mlpst	0.0342±0.0019	0.0705±0.0022	22.43±0.28	0.9114±0.0064	0.8689±0.0087	0.8775±0.0079
Unist	0.0326±0.0013	0.0689±0.0029	22.78±0.35	0.9161±0.0053	0.8806±0.0103	0.8862±0.0088
Openstl	0.0314±0.0018	0.0671±0.0025	23.11±0.31	0.9207±0.0046	0.8778±0.0095	0.8954±0.0069
V2xpnp	0.0301±0.0012	0.0656±0.0021	23.45±0.24	0.9249±0.0058	0.8915±0.0078	0.9026±0.0091
DFGNet	0.0287±0.0016	0.0632±0.0019	23.91±0.29	0.9305±0.0039	0.9064±0.0082	0.9147±0.0065
Ours	0.0254±0.0014	0.0578±0.0023	24.78±0.26	0.9349±0.0047	0.9315±0.0076	0.9382±0.0072

Table 3 presents the quantitative performance of different models on the training set. The proposed method achieves the best results across all six evaluation metrics, with the lowest MAE and RMSE of 0.0254 and 0.0578, respectively, and the highest PSNR, SSIM, NSE, and KGE values of 24.78, 0.9349, 0.9315, and 0.9382. These results indicate that the proposed framework has a stronger ability to fit the spatiotemporal patterns of runoff-field evolution in the training data. Compared with the baseline models, the improvement is not limited to pixel-level error reduction, but is also reflected in better spatial structural preservation and hydrological consistency. In particular, the higher NSE and KGE values show that the predicted runoff fields are more consistent with the observed fields in terms of overall variation, fluctuation magnitude, and statistical bias. Meanwhile, the standard deviations remain within a relatively stable range, suggesting that the proposed method maintains robust fitting performance across repeated runs. Combined with the testing results in Table 2, the training performance does not show an excessive gap from the testing performance, indicating that the model does not suffer from obvious overfitting while still maintaining sufficient representation capacity.

### 5.2 The ablation experiment results of the algorithm in this paper

To further clarify the contribution of each key component to the overall forecasting capability, we conduct an ablation study under the same training configuration and data split. Starting from the backbone, we progressively introduce the MLCG cross-scale gating mechanism and the BTA basin-topology attention module, and we validate the full model as the final combination. This ablation setting is designed to separately examine the role of cross-scale temporal fusion in error convergence and field-detail restoration, as well as the gains brought by injecting topological priors for modeling spatial structural consistency. We uniformly evaluate both numerical accuracy and structural fidelity using six metrics, namely MAE, RMSE, PSNR, and SSIM. The corresponding ablation configurations and comparison results are summarized in Table 4.

**Table 4:** Test-phase ablation study on key components.

Method	MAE	RMSE	PSNR	SSIM	NSE	KGE
Baseline	0.0412±0.0023	0.0846±0.0038	20.61±0.43	0.8794±0.0065	0.7876±0.0131	0.8069±0.0116
+MLCG	0.0367±0.0019	0.0769±0.0031	21.78±0.37	0.9014±0.0056	0.8489±0.0098	0.8617±0.0091
+BTA	0.0329±0.0016	0.0698±0.0027	22.86±0.32	0.9169±0.0045	0.8728±0.0085	0.8881±0.0078
Ours	0.0269±0.0011	0.0603±0.0019	24.39±0.22	0.9273±0.0038	0.9187±0.0069	0.9264±0.0063

Table 4 shows that each introduced component brings clear and complementary improvements. Compared with the Baseline, adding MLCG reduces MAE from 0.0412 to 0.0367 and RMSE from 0.0846 to 0.0769, while increasing PSNR from 20.61 to 21.78, SSIM from 0.8794 to 0.9014, NSE from 0.7876 to 0.8489, and KGE from 0.8069 to 0.8617, indicating that cross-scale temporal fusion effectively improves error control and hydrological consistency. After further introducing BTA, the model achieves 0.0329 MAE and 0.0698 RMSE, together with 22.86 PSNR, 0.9169 SSIM, 0.8728 NSE, and 0.8881 KGE, showing that basin-topology-guided spatial modeling further strengthens structural preservation and runoff variation characterization. When both modules are jointly used, the full model attains the best results on all metrics, with MAE 0.0269, RMSE 0.0603, PSNR 24.39, SSIM 0.9273, NSE 0.9187, and KGE 0.9264, which verifies the effectiveness and complementarity of combining cross-scale gating with basin-topology attention. Similarly, the training set results of the ablation experiments are presented in Table 5.

**Table 5:** Training-phase ablation study on key components.

Method	MAE	RMSE	PSNR	SSIM	NSE	KGE
Baseline	0.0387±0.0018	0.0795±0.0032	21.13±0.39	0.8902±0.0071	0.8124±0.0117	0.8265±0.0108
+MLCG	0.0341±0.0021	0.0726±0.0025	22.36±0.31	0.9113±0.0049	0.8697±0.0093	0.8794±0.0086
+BTA	0.0308±0.0014	0.0662±0.0029	23.42±0.34	0.9254±0.0052	0.8925±0.0076	0.9047±0.0091
Ours	0.0254±0.0014	0.0578±0.0023	24.78±0.26	0.9349±0.0047	0.9315±0.0076	0.9382±0.0072

Table 5 reports the training-phase ablation results of the proposed framework. Compared with the corresponding testing-phase results, the training performance is only slightly better, and the overall trend remains consistent across different component settings. This indicates that the improvements brought by MLCG and BTA are not caused by simply fitting the training samples, but reflect stable representation enhancement. The full model maintains the best performance in both the training and testing phases, while no excessive gap is observed between them, suggesting that the proposed framework does not suffer from obvious overfitting. Meanwhile, the consistently strong results on the training set also show that the model has sufficient fitting ability and does not exhibit underfitting. Therefore, the ablation results further verify that the proposed components improve forecasting capability while maintaining stable generalization.

### 5.3 Visualize experimental results

#### 5.3.1 Qualitative results compared with other models.

**Fig 6:**
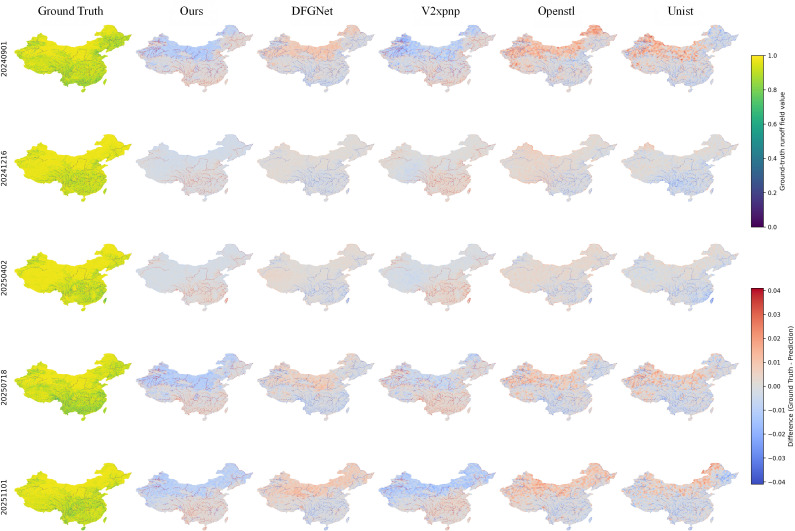
Qualitative comparison of spatial difference maps between the proposed method and representative competing models. The first column shows the ground-truth runoff fields, while the remaining columns present the corresponding difference maps between the ground truth and each model prediction under a unified color scale.

To more intuitively illustrate the differences among methods in recovering the spatial structure of runoff fields, we select several representative temporal samples from the test set and provide a qualitative visualization comparison between the predicted fields and the ground-truth fields. Considering both methodological representativeness and recent progress, we mainly choose DFGNet, V2X-PNP, OpenSTL, and UniST as baselines, and conduct a fair comparison under the same input window and forecasting horizon settings. The qualitative results are shown in Fig 6.

As shown in Fig 6, the first column provides the reference runoff-field patterns at different forecasting dates, while the remaining columns visualize the spatial forecasting differences of each model. The difference maps indicate that the proposed method produces more spatially coherent and lower-amplitude errors than the compared models, with most deviations concentrated along major runoff corridors and high-response regions rather than being broadly distributed over the whole domain. In contrast, DFGNet, V2xpnp, Openstl, and Unist exhibit more evident positive or negative deviations in several river-network and high-runoff areas, suggesting stronger local underestimation or overestimation. Since all difference panels share the same color scale, the comparison directly shows that the proposed method better suppresses spatial error propagation over time and preserves the structural consistency of runoff fields more effectively.

#### 5.3.2 Cross-sectional comparison experiment.

**Fig 7:**
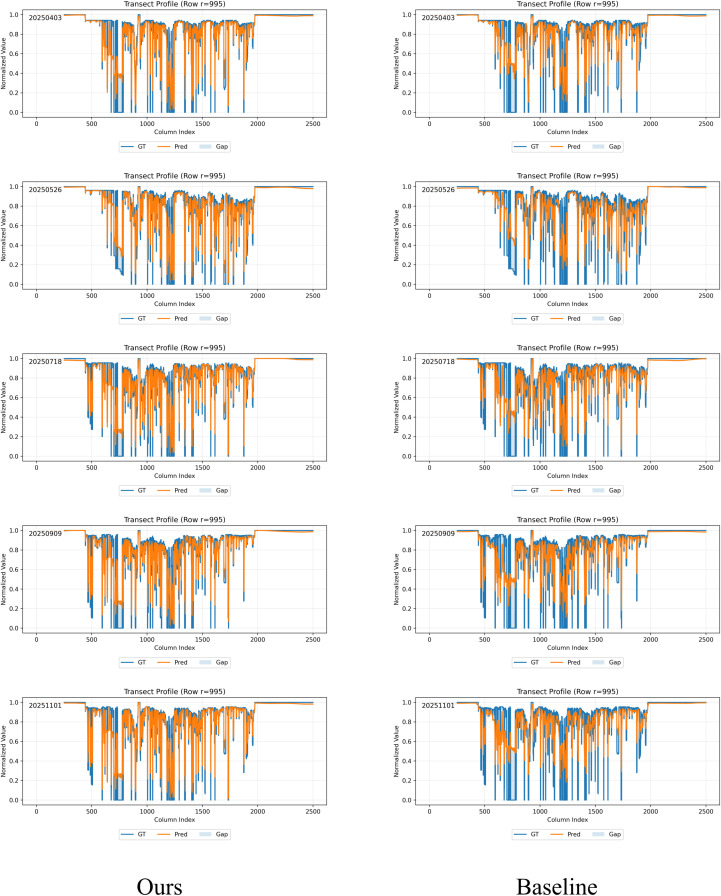
Cross-sectional 1D transect profile comparison along a fixed line in the test set (row *r* = 995), showing the ground truth (GT), predictions (Pred), and their difference (Gap) for Ours and the Baseline across multiple temporal samples to evaluate the modeling of spatial gradient variations and local extremes.

To further examine the model’s ability to capture spatial gradient variations and local extremes from a one-dimensional profile perspective, we conduct a cross-sectional profile visualization study and use only the Baseline as the reference model. Specifically, for test samples we fix the same spatial row or column as the profile line, unfold the 2D runoff field along this direction into a 1D sequence, and plot the ground-truth and predicted curves simultaneously. By comparing the locations of peaks and valleys, amplitude changes, and local-detail consistency of the profile curves, we can more directly assess the fitting stability of the proposed method in key structural regions and its error convergence characteristics. The qualitative results are shown in Fig 7.

The transect-profile comparisons show that, compared with the Baseline, the proposed method produces predicted curves that are more consistent with the ground-truth curves in overall trends for most samples. In particular, it better tracks the peak–valley location changes in high-frequency fluctuation intervals, indicating more stable modeling of spatial gradients and local structures. In contrast, the Baseline forecasting along the same transect are more prone to accumulated amplitude deviations and local over-oscillation, with noticeable misalignment at some valley locations, resulting in more continuously deviating segments along the profile. Overall, these observations suggest that cross-scale gating enhances the fusion of dynamic information across different scales, while the structural constraints introduced by topology attention help reduce systematic biases in key structural regions, thereby improving profile-level fitting consistency and robustness.

#### 5.3.3 Grad-CAM visualization results.

**Fig 8:**
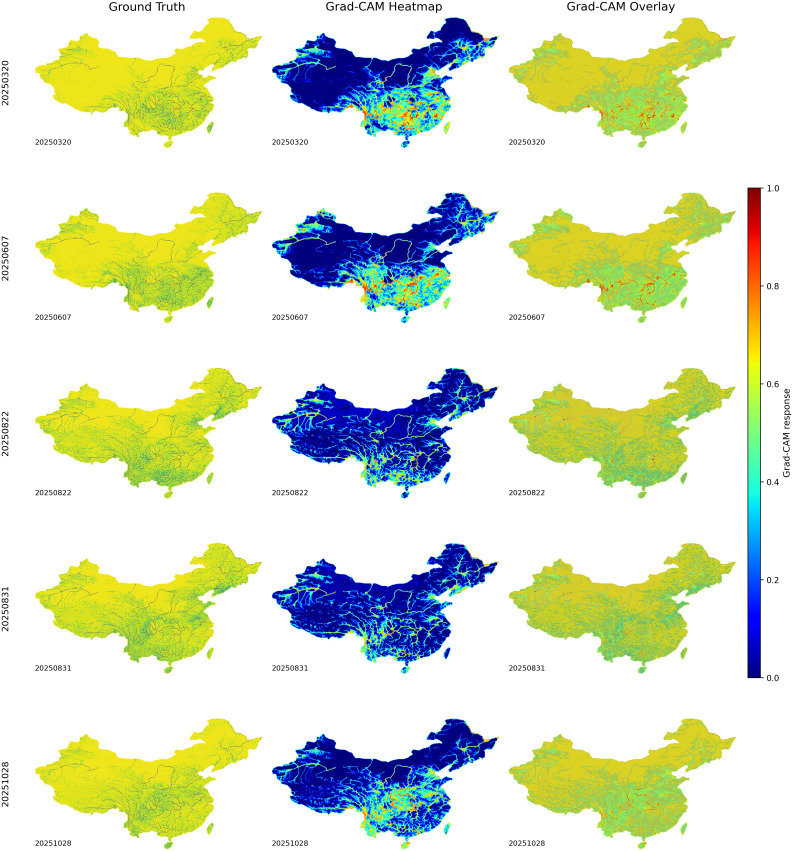
Experimental results of the grad-cam algorithm proposed in this paper.

To improve the interpretability of the model forecasting and analyze the spatial distribution characteristics of the attended regions, we perform Grad-CAM visualization for the proposed model. Based on the output response corresponding to the forecasting target, we compute gradient-guided class activation maps and overlay them on the input runoff fields to observe the attention intensity and focus locations across different regions. In this study, successful forecasting behavior is reflected by attention patterns that concentrate primarily on major river-network corridors, confluence regions, and spatial areas with high runoff variability, while maintaining relatively weak responses in stable background regions or invalid masked areas. Such a distribution indicates that the model captures the key hydrological structures and dynamically active regions most relevant to runoff evolution, rather than relying on diffuse or physically irrelevant responses. The qualitative results are shown in Fig 8.

The Grad-CAM results show clear spatial selectivity across different samples, with high-response regions mainly distributed along major river corridors, tributary confluence areas, and zones exhibiting stronger runoff variation, whereas relatively stable background areas remain weakly activated. This indicates that the model focuses its predictive evidence on hydrologically meaningful structural regions and dynamically sensitive areas, thereby supporting more spatially consistent and reliable runoff forecasting.

### 5.4 Hyperparameter sensitivity experimental results

To evaluate the stability and reproducibility of the proposed method under variations in training configurations, we further conduct a hyperparameter sensitivity analysis, focusing on the effects of the learning rate and optimizer choice on convergence behavior and forecasting performance. Specifically, while keeping the data split, input window, and network architecture unchanged, we repeatedly train and evaluate the model under different learning-rate settings and several commonly used optimizer configurations to examine the magnitude of performance fluctuations and the distribution of optimal regions. This experiment aims to verify the robustness of the model to key training hyperparameters and to provide more reliable configuration guidance for practical deployment. The results are reported in Table 6.

**Table 6:** Hyperparameter sensitivity results on learning rate and optimizer.

Setting	Value	MAE	RMSE	PSNR	SSIM
Learning rate	4e−4	0.0347±0.0018	0.0729±0.0031	22.71±0.34	0.9033±0.0053
	3e−4	0.0318±0.0015	0.0678±0.0028	23.42±0.29	0.9135±0.0049
	2e−4	0.0297±0.0013	0.0641±0.0024	23.96±0.25	0.9201±0.0044
	1e−4	0.0269±0.0011	0.0603±0.0019	24.39±0.22	0.9273±0.0038
Optimizer	AdaGrad	0.0351±0.0019	0.0733±0.0032	22.66±0.37	0.9011±0.0055
	SGD	0.0339±0.0017	0.0714±0.0029	22.91±0.33	0.9064±0.0051
	Adam	0.0308±0.0014	0.0662±0.0025	23.52±0.27	0.9178±0.0047
	AdamW	0.0269±0.0011	0.0603±0.0019	24.39±0.22	0.9273±0.0038

The learning-rate sensitivity comparisons reveal a clear performance gradient across different learning rates. As the learning rate is gradually reduced from $4\times 10^{-4}$ to $1\times 10^{-4}$, the error metrics consistently improve and PSNR and SSIM increase accordingly. This indicates that an excessively large learning rate is more likely to cause overly aggressive parameter updates and unstable convergence, which in turn degrades the modeling of spatial details and structural consistency. In contrast, $1\times 10^{-4}$ achieves a better balance between error reduction and structural fidelity, exhibiting a more stable optimization trajectory and more thorough convergence, which also aligns with the strong non-stationarity of this task and its sensitivity to slender structural details.

The optimizer sensitivity results further show that AdamW, which incorporates adaptive momentum and decoupled weight decay, outperforms AdaGrad, SGD, and Adam. This suggests that in high-dimensional spatiotemporal forecasting tasks, relying solely on accumulated gradients or fixed momentum can lead to an insufficient trade-off between convergence speed and generalization, whereas the weight-decay treatment in AdamW more effectively suppresses overfitting and improves structural fidelity. Overall, the two sensitivity studies jointly indicate that the model is reasonably robust to key training hyperparameters within a practical range, while also providing a more reliable default configuration that helps maintain consistent forecasting quality under different training conditions.

### 5.5 Representative basin analysis

To further examine the regional applicability of the proposed framework under different hydrological conditions, three representative basins in China are selected for focused analysis, namely the Yangtze River Basin, the Yellow River Basin, and the Pearl River Basin. The Yangtze River Basin is approximately cropped within longitude 90°E–122°E and latitude 24°N–35°N, the Yellow River Basin within longitude 96°E–119°E and latitude 32°N–42°N, and the Pearl River Basin within longitude 102°E–116°E and latitude 21°N–29°N. These three basins cover distinct hydrological regimes and river-network structures, thereby providing a more interpretable basis for evaluating the model under representative large-scale, northern semi-humid to semi-arid, and southern humid runoff conditions. The experimental results are shown in Table 7.

**Table 7:** Representative basin analysis results of the proposed method.

Basin	MAE	RMSE	PSNR	SSIM
Yangtze River Basin	0.0248	0.0561	24.96	0.9348
Yellow River Basin	0.0297	0.0648	23.71	0.9186
Pearl River Basin	0.0229	0.0527	25.43	0.9412

From the representative basin analysis results, the proposed method maintains stable forecasting performance across all three basins, indicating good regional adaptability under different hydrological conditions. Among them, the Pearl River Basin achieves the best overall performance, with the lowest MAE and RMSE and the highest PSNR and SSIM, suggesting that the model can better reconstruct runoff patterns in humid regions with relatively strong spatial continuity. The Yangtze River Basin also shows strong performance, while the Yellow River Basin is relatively more challenging, as reflected by higher errors and lower structural similarity, which is likely related to its more complex runoff variability and heterogeneous basin characteristics.

### 5.6 Spatial error characteristics analysis

**Fig 9:**
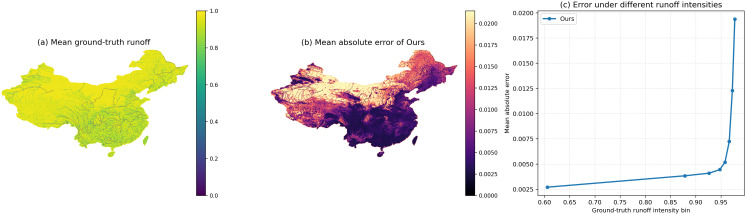
Spatial error characteristic analysis of the proposed method on the test set. Panel (a) shows the mean ground-truth runoff field, panel (b) presents the mean absolute error of the proposed method, and panel (c) illustrates the variation of mean absolute error across different ground-truth runoff intensity bins.

To further characterize the spatial distribution properties of model prediction errors, this study analyzes the relationship between runoff-field intensity and prediction error within the valid regions of the test set. The analysis is conducted from three perspectives: spatially averaged runoff, spatially averaged absolute error, and error variation across different runoff-intensity intervals. This provides a complementary description of regional error structures that are difficult to capture using overall evaluation metrics alone. The experimental results are shown in Fig 9.

As shown in Fig 9, the model prediction errors are not uniformly distributed in space, but are closely related to runoff intensity and hydrological spatial structure. Fig 9(a) presents the spatial distribution of the average ground-truth runoff field during the test stage, where clear regional differences in runoff intensity can be observed. Fig 9(b) further shows that the mean absolute error is mainly concentrated in regions with high runoff response, major runoff channels, and areas with strong spatial variation, whereas the errors in low-runoff regions remain relatively small overall. Fig 9(c) quantitatively confirms this phenomenon. As the ground-truth runoff intensity interval gradually increases, the mean absolute error also shows an overall upward trend, with a more pronounced increase in high-runoff intensity intervals. These results indicate that the difficulty of multi-step runoff field prediction is not determined solely by the overall temporal variation. Strong nonlinear responses, spatial flow-concentration effects, and local structural complexity in high-runoff regions also substantially increase prediction uncertainty.

## 6 Limitation

Although the GloFAS Historical dataset provides a consistent and operationally valuable basis for large-scale runoff-field forecasting over China, its relatively coarse spatial resolution may limit the representation of fine-scale hydrological variability within complex basins and sub-basin units. In particular, localized runoff responses in narrow tributaries, small confluence zones, and regions with strong terrain heterogeneity may be smoothed or only partially preserved at the current grid scale. Since the main objective of this study is to establish a China-scale gridded runoff forecasting benchmark and to evaluate the effectiveness of the proposed end-to-end spatiotemporal framework under a unified large-scale setting, the adopted resolution is sufficient for modeling broad spatial patterns, major river-network structures, and multi-step runoff evolution. Under the current experimental design, the superiority of the proposed model over the compared methods has been verified within the adopted multi-step forecasting horizon of *Q* = 7, whereas whether this advantage can be consistently maintained over longer lead times still requires further dedicated investigation in future work. Nevertheless, for applications requiring finer sub-basin characterization, future work may further incorporate spatial downscaling strategies, such as statistical downscaling or learning-based super-resolution methods, to enhance the reconstruction of local runoff dynamics and improve the representation of small-scale hydrological details.

The current study is conducted under a data-driven runoff-field forecasting setting based on gridded runoff fields derived from the publicly available GloFAS Historical dataset, rather than under a fully meteorology-driven or process-based hydrological modeling framework with explicit external forcings and basin parameterization. We acknowledge that precipitation is a fundamental driver of runoff generation and that several gridded precipitation products are available for hydrological applications. However, the objective of this study is to evaluate whether historical runoff-field sequences, combined with cross-scale temporal modeling and basin-topology-aware spatial representation, can support multi-step runoff-field forecasting under a unified runoff-only benchmark. Therefore, precipitation, evapotranspiration, temperature, soil moisture, and other external hydrometeorological forcing variables are outside the scope of the present study and are not incorporated into the current experimental design. Accordingly, classical physics-based hydrological models are not included in the present comparison, because such models usually require a complete set of meteorological forcings, basin descriptors, calibration procedures, and routing parameters that are beyond the runoff-sequence forecasting setting considered here. The present evaluation therefore focuses on the relative effectiveness of the proposed method against representative data-driven baselines under the same gridded runoff-field forecasting protocol. Future work will extend the framework by incorporating precipitation and other hydrometeorological drivers, together with representative basin-level case studies, to enable more comprehensive comparisons with physics-based hydrological models under different hydrological regimes.

## 7 Conclusion

This study investigates the task of multi-step runoff-field forecasting over China and develops an end-to-end spatiotemporal forecasting framework that integrates cross-scale gating with basin-topology attention. Based on the public GloFAS Historical dataset and a unified experimental protocol, the proposed method consistently achieves the best overall performance among all compared models, reaching MAE 0.0269, RMSE 0.0603, PSNR 24.39, SSIM 0.9273, NSE 0.9187, and KGE 0.9264. These results indicate that the framework not only reduces numerical prediction errors, but also better preserves spatial structural consistency and more faithfully captures the hydrological variation characteristics of runoff fields. The ablation results further show that cross-scale gating mainly improves the modeling of multi-timescale temporal dynamics, while basin-topology attention provides additional gains in spatial dependency characterization and structural fidelity; when jointly incorporated, the two modules yield clear complementary benefits across all evaluation metrics.

The main contribution of this study lies in providing a structured deep-learning solution for China-scale runoff-field forecasting that explicitly combines temporal cross-scale interaction and river-network topology guidance within a unified framework. Different from conventional data-driven forecasting models that mainly rely on generic temporal encoders or spatial aggregation, the proposed method introduces hydrologically meaningful inductive biases into both temporal fusion and spatial dependency learning, thereby improving forecasting accuracy, structural coherence, and hydrology-oriented consistency at the same time. In addition, this study constructs a reproducible gridded runoff forecasting benchmark with corresponding valid-region masking and standardized evaluation settings, which provides a practical basis for subsequent model comparison and methodological extension. Overall, the proposed framework contributes to bridging general spatiotemporal deep learning with hydrology-aware structural modeling, and offers a feasible approach for regional runoff prediction, flood-risk analysis, and intelligent hydrological forecasting applications.

Future work will extend this framework from both the data and modeling perspectives. On the data side, finer-grained river-network priors, sub-basin descriptors, and more diverse hydrometeorological driving factors can be incorporated to further improve the representation of localized runoff evolution and complex hydrological responses. On the modeling side, more efficient topology-aware dependency learning and lightweight high-resolution forecasting strategies will be explored to reduce computational overhead and improve adaptability in real-time forecasting and operational deployment scenarios.
